# New Method of Production and Characterization of Haemozoin and B-Haemozoin from *Meccus longipennis*

**Published:** 2019

**Authors:** Liliana GONZÁLEZ-LINARES, Víctor Esteban REYES-CRUZ, María Aurora VELOZRODRÍGUEZ, Gustavo URBANO-REYES, José Luis IMBERT-PALAFOX, José Angel COBOS-MURCIA

**Affiliations:** 1. Department of Medicine, School of Biological and Health Sciences, Autonomous University of Hidalgo State, Hidalgo, México; 2. Department of Materials and Earth Sciences, School of Engineering and Basic Sciences, Autonomous University of Hidalgo State, Hidalgo, México; 3. Mexican National Council for Science and Technology, México City, México

**Keywords:** Haemozoin, *Meccus longipennis*, Drug evaluations, β-haemozoin, Dimerization, Malaria

## Abstract

**Background:**

Understanding the significance of hemozoin (Hz) in the process through which *Plasmodium* is released from the heme group in the food vacuole during hemoglobin degradation, will allow the development of more effective drugs against malaria. Therefore, the development of methodologies to obtain Hz synthetically will facilitate an in vitro evaluation of new anti-malarial drugs.

**Methods:**

We present a methodology with good results to obtain Hz from fecal material of blood-sucking insects *Meccus longipennis*. The preparation of biological cultures of the parasite (*Plasmodium*) transmitter of the disease is not necessary.

**Results:**

The hemozoin molecule and its dimer were obtained using the method described and it was possible to validate a comparison with the positive and negative controls using different analytical techniques.

**Conclusion:**

The proposed method allows obtaining hemozoin and its dimer demonstrating equivalence with positive controls that demonstrate that the present procedure may be an alternative for the evaluation of antimalarial drugs.

## Introduction

Malaria remains a public health problem worldwide. WHO estimates that each year there are 212 million new cases and even 429 thousand deaths worldwide (1). Malaria is caused by a protozoan parasite of the genus *Plasmodium* hematologic, being *P. falciparum* the deadliest species. This parasite is developed through a complex life cycle happening between the vector, the female *Anopheles* mosquito (sporogonic cycle) and the human host. In the latter, the parasite goes through two consecutive cycles; an exoerythrocytic cycle within liver cells and an erythrocytic cycle, which infects red blood cells and degrades hemoglobin because the parasite feeds itself of hemoglobin to obtain from it the necessary amino acids to synthesize its own proteins (2, 3).

The free heme group obtained from degradation of hemoglobin, also known as heme or hidroxiferriprotoporfirina IX, is very toxic because it can generate oxygen reactive species. To avoid the toxicity of the free heme group, the parasite has developed some detoxification systems (3–7). Such systems can be carried out in an acid digestive vacuole or in the cytoplasm, converting the free heme group in hemozoin (Hz) through a mechanism known as Hz formation. This mechanism is not very clear, however, this process is generally accepted as a biomineralization or biocrystallization (3, 8).

On the other hand, Oliveira et al. (9–11) and Wood et al. (12) identified the production of the free heme group in other blood-sucking organisms, such as *Rhodnius prolixus*, the helminth *Schistosoma mansoni*, main etiologic agent of schistosomiasis and protozoan *Haemoproteus columbae*. In these organisms, the formation of Hz is the main detoxification way of the free heme group by the perimicrovillar membranes. They showed that the structural and morphological characterization of free heme crystals, produced by *R. prolixus* and *S. mansoni,* provides important information to develop new drugs against Chagas disease and schistosomiasis.

In the case of malaria, it is important to perform studies about the process to obtain the Hz, its structure and its effects within the cell, that provide information to develop new drugs that are more effective and with less toxic effects.

In this regard, there are research works on obtaining Hz from microbe cultures of different species of *Plasmodium* on human erythrocytes using an excessive number of parasites and complicated processes to obtain substantial amounts of Hz (12, 13). Other methods use lipid systems like the system within the vacuole, from Hz obtained from Plasmodium used to grow Hz (14) crystals. While non-biological methods, in spite of having a simple preparation, have low yields since the amount of obtained Hz is less than 50% of total reagent employed (12, 15).

Synthetic hemozoin that presents the best advantages employ haemozoin biocrystallization pathway and have been used successfully in the evaluation of antimalarial drugs (13, 16), so that different studies have been conducted to evaluate the difference between the natural and syntactic β-Hz, showing the similarities between both (17).

Regarding the characterization of Hz molecules and its dimer (β-Hz), obtained by various processes, there have been several researches. The UV-visible spectroscopy characterization (18), NRM (19), IR (20), and XRD (21) have demonstrated that the malarial Hz and its synthetic homologue are identical do to the characteristic bands of absorbance they have, such as the Soret band in UV-Vis and the typical vibrations in the infrared region associated with the bond between iron and oxygen during dimer formation (18). Studies have been conducted using computational methods to determine: a) the most probable structures of Hz and β-HZ, and b) the obtention by simulation of IR spectra, which confirm the structural models proposed for Hz and its dimer (22) with experimental results. On the other hand, acidity constants and dimerization of Hz using spectroscopic techniques (23) have been determined.

Therefore, the present methodology also has the advantage that the method of feeding, defecation and development times of *Meccus longipennis* in the laboratory (24), is well studied and standardized. Because this parasite is the main transmission vector of *Trypanosoma cruzi*, which causes Chagas disease (25). As the obtaining with this insect can be controlled, you facilitate the study of Hz and β-Hz molecules. Furthermore, the characterization of is done through different analytical techniques.

## Materials and Methods

### Biological material

The colonies of *Longipennis* were maintained in wide-mouth jars, at 29 ± 2°C and 70% relative humidity. The insects were fed weekly on immobilized mice of the mus muscle species.

### Obtention and preparation of the Hz and β-Hz from M. Longipennis

Insects’ feces and urine adhered to the filter paper were collected and pulverized. All this material is placed in a container continuously stirred with a magnetic stirrer for 24 hours in absolute ethanol. The mixture was filtered using a cellulose membrane and subsequently dialyzed with four changes of 2 litters of water at 4 °C for 24 hours. Then, 100 mL of sodium azide (0.01 mM) were added from the beginning, and at the last dialysis, EDTA acid was added. The dialyzed material was moved to conical tubes left at rest at a temperature of 4°C for 24 hours. The conical tubes were centrifuged at 2,500 revolutions per minute (rpm) for 20 minutes, the supernatant was filtered and later it was mixed with 1M NaOH. β-Hz is obtained at this point.

The β-Hz solution was acidified with concentrated HCl (13.87 M), it was left precipitating for another 24 hours, then the supernatant was discarded, and the precipitate was transferred to conical tubes to centrifuge at 2,500 rpm for 20 minutes. The precipitate was washed with deionized water (pH= 6) between five and eight times and the last wash was stirred at 200 rpm at room temperature (25 °C) for 24 hours. This washing procedure was performed for three consecutive days and finally the precipitate was separated and dried at 32 °C to obtain Hz from *Longipennis* (26).

Finally, the purification was carried out by washing the crystals with deionized water, it was buffered to pH 6, it was centrifuged and the supernatant was removed, the washing was repeated seven times and it was placed under agitation at a temperature of 25 °C, for 24 hours. This purification process was repeated every day until all uric acid residues were eliminated from the insect’s urine, monitoring the presence of crystals of this impurity using microscopy.

### Positive and negative controls

Swine hematite (SH) from Becton & Dickinson was used as positive control and protoporphyrin IX (PP) from Sigma-Aldrich as negative control. These molecules were characterized using the same analytical techniques and were selected to differentiate the presence and absence of iron, in the SH and PP analyses, respectively. That compared to the use of control molecules of Hz and β-Hz (17, 26) it is only possible to analyze the difference due to the formation of the dimer. All the described reagents were analytical grade and the solutions were prepared using deionized water with 18 M MΩ.

### Characterization by Scanning Electron Microscopy (SEM)

An electronic scanning microscope JEOL model 6300 was used to obtain scanning electron micrographs (SEM) and the spectra of electro dispersed electrons (EDS) of the molecules Hz and β-Hz from *Longipennis*. The microscope was equipped with an energy dispersive spectrometer (EDS), using 5000X magnification to observe topographic images and a secondary electron energy of 30 kV for ESD.

### Characterization by X-Ray Diffraction (XRD)

An Inel equipment, model Equinox 2000, was used to obtain XRD data of Hz and β-Hz both from *Longipennis*, SH and PP. Analyses were carried out in the range of 0 to 120° measured using cobalt radiation (CoKα1).

### Characterization by FT-IR spectroscopy.

Samples of Hz and β-Hz both from Longipennis, SH and PP were pulverized and used to make KBr tablets. They were swept in a wavelength range of 370–4000 cm^−1^ and 650–4000 cm^−1^ FTIR using Attenuated Transmittance Reflectance system in a ZnSe crystal. To perform this study, a Spectrum v.3.02 software was used.

### Characterization by UV-Vis spectroscopy

A 10 mM solution in NaCl 150 mM of Hz and β-Hz, both from *Longipennis*, SH and PP, was prepared to carry out a study of UV-visible in a range of 300–700 nm. A Perkin-Elmer UV-visible absorption equipment model Lambda 2s was used.

### Characterization by Zeta Potential

A sample of Hz from *Longipennis* was dissolved in deionized water at a concentration of 0.1M, and five adjustments of pH values of 4, 5, 6, 8 and 9 were made to study the zeta potential in a range of 0 to −35 mV. This was possible by using a Zetasizer Malvern model 3000 HAS equipment.

## Results

### Obtaining Hz from Longipennis

2.61 g were collected from faeces and urine samples by using filter paper sheets. They helped obtaining 1.02 g of Hz, which represent 39% of the original sample.

### SEM and EDS

**Fig. 1:**
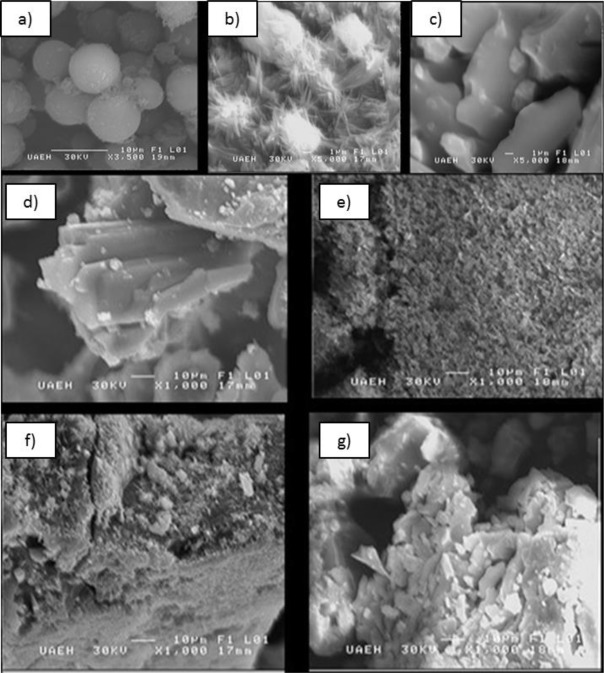
Micrographs of a) Faeces of *M. longipennis*, b) Hz, c) β-Hz, c) SH as a positive control, d) PP as a negative control e) Hz and f) β-Hz from M. longipennis, using secondary electrons of 30 KV, with 3,500 and 5,000X increases

Fig. 1 shows the micrographs of a) Feces of *Longipennis*, b) Hz and c) β-Hz, obtained from *Longipennis*; obtained by using the purification methodology previously described. Micrographs of d) Swine as a positive control and e) PP; as a negative control can be seen, and comparatively, micrographs of f) Hz and g) β-Hz from *Longipennis* that were are obtained. Using secondary electrons to 30 KV, with 3,500 and 5,000X increases respectively.

EDS studies performed to these same molecules show signals of Fe present in the central part of Hz and in β-Hz molecules. From the EDS results, it is obtained Table 1 on the composition of Hz and β-Hz from *Longipennis*, SH and PP.

**Table 1:** Chemical composition of molecules evaluated with Energy-dispersive X-ray spectroscopy

***Element***	***Hz M. longipennis (Wt %)***	***β-Hz M. longipennis (Wt %)***	***SH Positive control (Wt%)***	***PP Negative control (Wt%)***
Iron	7.62 ± 7.52	28.86 ± 3.79	9.98 ± 0.19	0.0 ± 0.0
Carbon	42.25 ± 1.40	29.47 ± 1.0	57.58 ± 0.62	29.47 ± 0.72
Nitrogen	19.0 ± 9.85	17.84 ± 3.01	13.62 ± 3.91	13.45 ± 5.23
Oxygen	31.13 ± 3.79	23.84 ± 2.05	15.52 ± 1.66	21.98 ± 1.28

### FTIR spectroscopy

**Fig. 2 :**
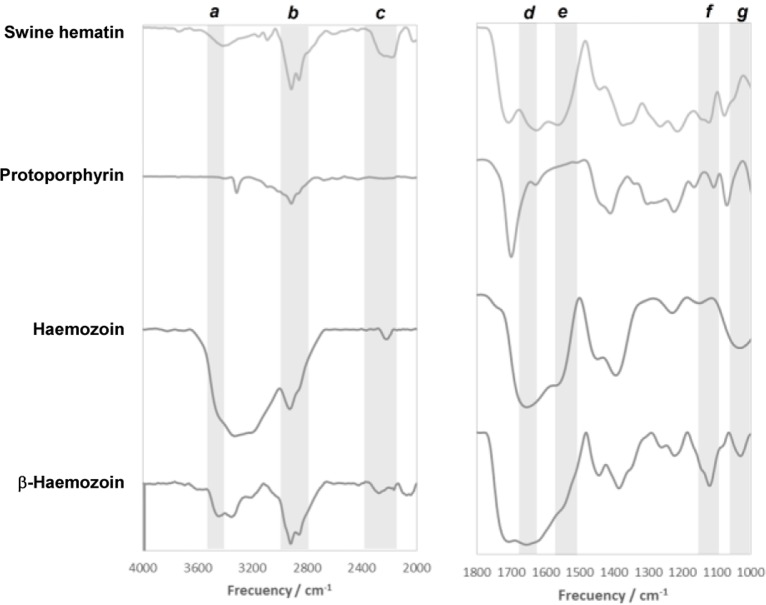
FTIR spectrum of Hz, β-Hz, PP and SH. a) Hz and b) β-Hz from *M. longipennis* c) SH as a positive control and d) PP as a negative control

Fig. 2 shows FTIR spectra of Hz and β-Hz from *M. longipennis* in a range of 1000–4000 cm^−1^. In which it can be seen the bands corresponding to carbonyl (C=O) and carboxylate groups (C-O); for Hz they are at 1671 and 1127 cm^−1^ (d and f regions respectively), while for β-Hz C=O was found at 1651 and C-O at 1046 in cm^−1^ (d and g regions respectively). In positive and negative controls, a series of bands associated to the vibration frequency of C=O and C-O bonds can be seen; which coincide with FTIR reports from literature (15, 21, 27).

### UV-Vis Spectroscopy

**Fig. 3:**
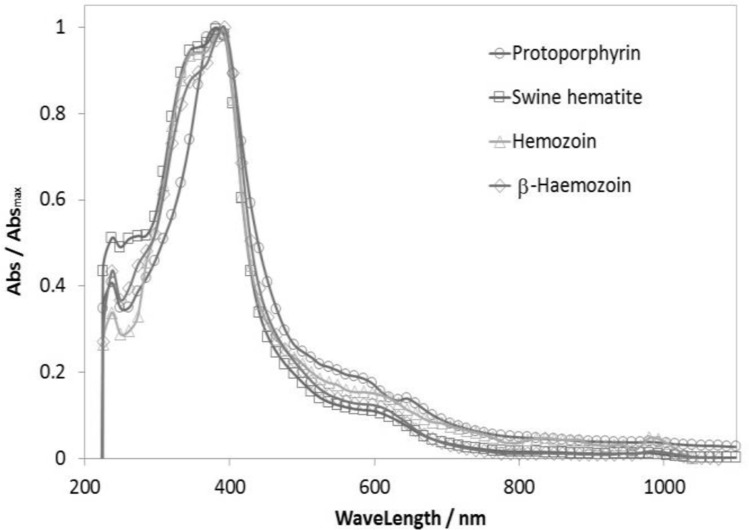
UV-Vis spectrum of Hz and β-Hz from M. *longipennis*, SH as a positive control and PP as a negative control

Fig. 3 shows the normalized UV-Visible absorption spectrum of Hz, β-Hz, positive and negative controls. In all cases, it is possible to observe the Soret band with a maximum absorption at 384, 391, 386 and 380 nm, respectively.

### X-ray diffraction

**Fig. 4:**
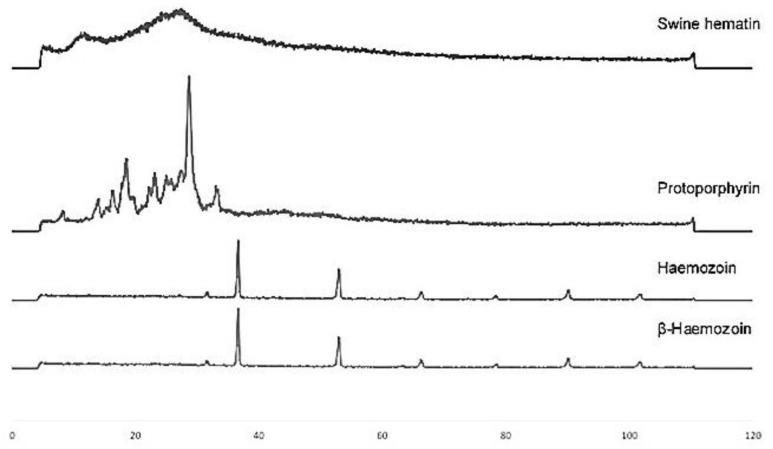
XRD pattern of Hz and β-Hz from *M. longipennis*, SH as a positive control and PP as a negative control

In Fig. 4 diffraction patterns of Hz, β-Hz, and PP, SH are shown in a range of 0 to 120 °, measured with Cobalt radiation (CoKα1). The obtained diffraction pattern signals of Hz and β-Hz from Longipennis, indicate the presence of a crystal structure of FCC type (Face Centre Cubic) with a Miller index [(1,1,1), (2,0,0), (2,2,0) and (3,1,1)].

### Zeta Potential

The zeta potential for dimerization of Hz by changing the pH value from 4 to 9 in a range of 0 to −35 potential mV; as well as the first derivative of zeta potential with respect to pH value.

## Discussion

In the micrographs of feces, spherical agglomerates can be observed (Fig. 1a), which are attributed to fecal matter with a particle size of 10 μm. In Fig. 1b, agglomerates with needle shape in different particle sizes can be seen, in the order of 3μm in the Hz sample. While in the β-Hz sample (Fig. 1c) crystalline agglomerates with homogeneous particle size of 10 μm can be observed. Both purified samples, present no traces of uric acid or faecal matter of *Longipennis*, which validates the established purification method to obtain haematin.

In Fig. 1c, crystalline morphology is observed with particle size of 50 μm, which is associated to the prosthetic group. While, in Fig. 1d, amorphous agglomerates with particle size of 1μm which also associated with prosthetic group, are observed. When comparing samples of Hz y β-Hz (Fig. 1f and 1g), it can be seen that the morphology of these samples is different from the positive (Fig. 1d) and negative (Fig. 1e) controls.

The peaks associated with gold (Au) in EDS are due to the coating of the sample, while, the peaks associated with chlorine (Cl) are due to traces of the solutions used during the purification process. On the other hand, the presence of Fe in the prosthetic group can also be appreciated in the positive control; whereas in the negative control there is no signal of Fe in EDS. It is noted that the composition of Fe in β-Hz is 3.8 times higher than in Hz. Demonstrating dimer obtaining with the purification methodology employed. While, the positive control (SH) contains a similar proportion to Hz of Fe (7.62 and 9.98 Wt.%, respectively) because chemically it is the same molecule. On the other hand, in the negative control (PP), there are no signals associated with Fe because this molecule does not contain it.

Furthermore, the band in FT-IR studies that corresponds to the stretching vibration of the Fe-N bond (reported for the formation of metal complexes) presents transmittance peaks (region c) of 2160 to 2315 cm^−1^ (28). Observing the same vibration in β-Hz at 2275 cm and a shoulder at 2310 cm^−1^, while Hz is 2318 cm^−1^. The band of the Fe-N bond of the positive control (SH) is at 2181 and 2247 cm^−1^ and it is not possible to see this band in the negative control (PP). Also, this same vibration has another band between 1573 and 1555cm^−1^ (region e); which is seen in all molecules except in the negative control and coincides with literature (27).

The SH and Hz present a band between 3440 and 3450 cm^−1^ (region a), which corresponds to the vibration of Fe-O bond, coupled with ^−^OH. This vibration is also present for β-Hz, however, in this case it is associated to the C-O bond of the dimer formation. The negative control (PP), does not present this peak as this molecule does not contain iron. Finally, in the range of 2800–3000 cm^−1^ (region b) there are bands that correspond to ^−^OH of the carboxyl groups present in all molecules of the prosthetic group, being of lower intensity for the negative control, due to the absence of this functional group (^−^OH) bound to Fe. These results validate the obtention of Hz and β-Hz from *Longipennis*

While in UV-Vis studies its show the similarity in the absorbance bands at a range of 345 to 390 nm of Hz and SH corroborates the presence of the same molecule. PP presents a hypsochromic shift at the band of maximum absorbance because iron is not bounded to the molecule, which favors progressive disruption of the electron system π as described in literature (29). In addition, it is observed the absence of absorbance bands in a wavelength of 345 nm, associated to the strong attraction that electrons suffer because of iron (30).

On the other hand, β-Hz presents a bathochromic shift in the maximum absorbance peak, with an offset of 7 nm and a decrease in the Soret band (360 nm) regarding Hz. This is consistent with previously reported for the formation of dimers and their respective equilibrium dimerization (18, 23).

In both molecules it is observed in X-ray diffraction the presence of the reflection peaks corresponding to the aromatic structure (00-004-0258) at 33.75, 38.85 and 55.18°; as well as peaks associated with Fe_3_N (01-073-2101) at 80.5, 92 y 103.56°. These results indicate that, the degree of crystallinity is similar, therefore they confirm the purity of the samples and that the procedure is suitable to obtain Hz from β-Hz coming from the *Longipennis.*

Moreover, the diffractograms responses of the positive and negative controls have signals in 4.5 to 3.5 corresponding to amorphous material typical of organic matter. It is appreciated that SH and PP have broad peaks indicating that these have a smaller particle size with respect to the β-Hz and Hz; which have peaks with a higher resolution and therefore a larger size.

In the studies of potential zero, at pH 6 an inflection point associated with the surface charge of the Hz molecule, starting dimerization in more alkaline pH; which is the result of the deprotonation suffered by the dimer at pH 6 and is consistent with literature regarding the dimerization process at pH = 6.2 ± 0.3 according with De Villiers (23). The information on the derivative of *dZ/dpH* confirms the change in slope of the inflection point at pH 6 by presenting a maximum peak at this pH value. Also, it is not observed any process associated to the deprotonation of monomer happening at pH 7.3 because the specie is already dimerized and deprotonated from a pH value of 6. This information indicates that to obtain Hz, pH values lower than 6 are required; as it occurs in the parasite’s food vacuole whose pH is 5.0 to 5.4 (31).

## Conclusions

The results of this study show an efficient, safe and effective methodology at a very low cost, to obtain Hz and β-Hz from a blood-sucking insect called *Longipennis*, since maintaining insects does not pose a risk of infection from malaria parasite, and it does not involve complicated procedures. In addition, the several analytical techniques used to identify β-Hz and Hz obtained from *Longipennis* demonstrate their correct obtention, as well as their purity. This was verified by simultaneously characterizing positive and negative controls; SH and PP respectively.

Due to the importance of Hz in the development of new antimalarial drugs, this paper provides the necessary information to purify Hz from fecal matter of hematophagous insects with good results, without being necessary the preparation of biological cultures of the parasite that transmits *Plasmodium* disease.

## References

[1] Word Health Organization (WHO) World malaria report. WHO Library Cataloguing, ISBN: 978 92 4 151171 1; 2016

[2] Fitch CD. Ferriprotoporphyrin IX, phospholipids and the antimalarial actions of quinoline drugs. Life Sci. 2004; 74: 1957–72. 14967191 10.1016/j.lfs.2003.10.003

[3] Sullivan DJ. Hemozoin: a biocrystal synthesized during the degradation of hemoglobin Biopolymers 9: Wiley online Library; 2005.

[4] Bendrat K Berger BJ Cerami A. Haem polymerization in malaria. Nature. 1995; 378: 138–9. 7477315 10.1038/378138a0

[5] Bohle DS Dinnebier RE Madsen SK Stephens PW. Characterization of the products of the heme detoxification pathway in malarial late trophozoites by X-ray diffraction. J Biol Chem. 1997; 272: 713–6. 8995354 10.1074/jbc.272.2.713

[6] Egan TJ. Haemozoin formation. Mol Biochem Parasitol. 2008; 157, 127–36. 18083247 10.1016/j.molbiopara.2007.11.005

[7] Stiebler R Hoang AN Egan TJ Wright DW Oliveira MF. Increase on the initial soluble heme levels in acidic conditions is an important mechanism for spontaneous heme crystallization in vitro. PLoS One 5 2010 5(9):e12694. 20856937 10.1371/journal.pone.0012694 PMC2938344

[8] Egan TJ Mavuso WW Ncokazi KK. The mechanism of beta-hematin formation in acetate solution. Parallels between hemozoin formation and biomineralization processes. Biochemistry. 2001; 40: 204–13. 11141072 10.1021/bi0013501

[9] Oliveira MF Silva JR Dansa-Petretski M De Souza W Lins U Braga CM. Haem detoxification by an insect. Nature 1999; 400: 517–8. 10448851 10.1038/22910

[10] Oliveira MF D'Avila JC Torres CR Oliveira PL Tempone AJ Rumjanek FD. Haemozoin in *Schistosoma mansoni*. Mol Biochem Parasitol. 2000; 111: 217–21. 11087932 10.1016/s0166-6851(00)00299-1

[11] Oliveira MF Kycia SW Gomez A Structural and morphological characterization of hemozoin produced by *Schistosoma mansoni* and *Rhodnius prolixus*. FEBS lett. 2005; 579: 6010–6. 16229843 10.1016/j.febslet.2005.09.035

[12] Wood BR Langford SJ Cooke BM Glenister FK Lim J McNaughton D. Raman imaging of hemozoin within the food vacuole of *Plasmodium falciparum* trophozoites. FEBS lett. 2003; 554: 247–252. 14623074 10.1016/s0014-5793(03)00975-x

[13] Thomas V Góis A Ritts B Burke P Hänscheid T McDonnel G. A Novel Way to Grow Hemozoin-Like Crystals In Vitro and Its Use to Screen for Hemozoin Inhibiting Anti-malarial Compounds. PLoS ONE 2012; 7: e41006. 22815894 10.1371/journal.pone.0041006 PMC3399802

[14] Ambele MA Sewell BT Cummings FR Smith PJ Egan TJ. Synthetic Hemozoin (β-Hematin) Crystals Nucleate at the Surface of Neutral Lipid Droplets that Control Their Sizes. Cryst Growth Des. 2013; 13(10): 10.1021/cg4009416. 10.1021/cg4009416 PMC3826461 24244110

[15] Slater AF Swiggard WJ Orton BR An iron-carboxylate bond links the heme units of malaria pigment. Proc Natl Acad Sci U S A. 1991; 88(2): 325–9. 1988933 10.1073/pnas.88.2.325 PMC50803

[16] Tempera T Franco R Caro C André V Eaton P Burke P Hänscheid T. Characterization and optimization of the haemozoin-like crystal (HLC) assay to determine Hz inhibiting effects of anti-malarial compounds. Malar J. 2015; 14: 403. 26458401 10.1186/s12936-015-0913-y PMC4603294

[17] Carvalho PA Coelho L Martins RC F. Nogueira F. Differences between synthetic β-haematin and native hemozoin crystals. Microscopy and Microanalysis. 2013; 19.

[18] Cole KA Ziegler J Evans CA Wright DW. Metalloporphyrins inhibit b-hematin (hemozoin) formation. J Inorg Biochem. 2000; 78: 109–15. 10766333 10.1016/s0162-0134(99)00216-0

[19] Gossuin Y Ndjolo PO Vuong QL Duez P. NMR relaxation properties of the synthetic malaria pigment β-hematin. Sci Rep. 2017; 7: 14557. 29109553 10.1038/s41598-017-15238-3 PMC5674059

[20] Wood BR Langford SJ Cooke BM Resonance Raman spectroscopy reveals new insight into the electronic structure of beta-hematin and malaria pigment. J Am Chem Soc. 2004; 126: 9233–9239. 15281812 10.1021/ja038691x

[21] Pagola S Stephens PW Bohle DS Kosar AD Madsen SK. The structure of malaria pigment beta-haematin. Nature 2000; 404: 307–10. 10749217 10.1038/35005132

[22] Marom N Tkatchenko A Kapishnikov S Kronik L Leiserowitz L. Structure and Formation of Synthetic Hemozoin: Insights From First-Principles Calculations. Cryst Growth Des. 2011; 11: 3332–41.

[23] De Villiers KA Kaschula CH Egan TJ Marques HM. Speciation and structure of ferriprotoporphyrin IX in aqueous solution: spectroscopic and diffusion measurements demonstrate dimerization, but not mu-oxo dimer formation. J Biol Inorg Chem. 2007; 12: 101–17. 16972088 10.1007/s00775-006-0170-1

[24] Martínez-Ibarra JA Grant-Guillén Y Martínez-Grant DM. Feeding, defecation, and development times of Meccus longipennis Usinger, 1939 (Hemiptera: Reduviidae: Triatominae) under laboratory conditions. Mem Inst Oswaldo Cruz. 2003; 98: 899–903. 14762515 10.1590/s0074-02762003000700007

[25] Martínez-Ibarra JA Grant-Guillén Y Morales-Corona ZY Importance of Species of Triatominae (Heteroptera: Reduviidae) in Risk of Transmission of *Trypanosoma cruzi* in Western Mexico. J Med Entomol. 2008; 45: 476–482. 18533443 10.1603/0022-2585(2008)45[476:iosoth]2.0.co;2

[26] Reyes-Cruz VE Reyes-Urbano G Veloz-Rodríguez MA Imbert-Palafox JL. Analysis of the electrochemical reactivity of natural hemozoin and β-hemozoin in the presence of anti-malarial drugs. Electrochimica Acta 2011, 9762–9768.

[27] Egan TJ Ross DC Adams PA. Quinoline anti-malarial drugs inhibit spontaneous formation of beta-haematin (malaria pigment). FEBS Lett. 1994; 352: 54–7. 7925942 10.1016/0014-5793(94)00921-x

[28] Davidson G. 2010 Chapter. 3: Vibrational spectra of transition element compound. En Spectroscopic Properties of Inorganic and Organometallic Compounds. Ed. The Royal Society of Chemistry; 2010.

[29] Bilia AR Lazari D Messori L Taglioli V Temperini C Vincieri FF. Simple and rapid physico-chemical methods to examine action of antimalarial drugs with hemin Its application to *Artemisia annua* constituents. Life Sci. 2002; 70: 769–78. 11833740 10.1016/s0024-3205(01)01447-3

[30] Majumdar S Mehdi OK Mitra S. Stability and characterization of Iron (III) and Iron (II) heme peptides encapsulated in aqueous detergent micelles: 1H NMR and UV-Vis spectroscopic studies. Inorganic Chem. 1991; 30: 700–5.

[31] Goldberg DE Slater AF Cerami A Henderson GB. Hemoglobin degradation in the malaria parasite *Plasmodium falciparum*: an ordered process in a unique organelle. Proc Natl Acad Sci. 1990; 87: 2931–5. 2183218 10.1073/pnas.87.8.2931 PMC53807

